# α-Actinin-2 Mediates Spine Morphology and Assembly of the Post-Synaptic Density in Hippocampal Neurons

**DOI:** 10.1371/journal.pone.0101770

**Published:** 2014-07-09

**Authors:** Jennifer L. Hodges, Samuel Martin Vilchez, Hannelore Asmussen, Leanna A. Whitmore, Alan Rick Horwitz

**Affiliations:** Department of Cell Biology, University of Virginia School of Medicine, Charlottesville, Virginia, United States of America; Northwestern University Feinberg School of Medicine, United States of America

## Abstract

Dendritic spines are micron-sized protrusions that constitute the primary post-synaptic sites of excitatory neurotransmission in the brain. Spines mature from a filopodia-like protrusion into a mushroom-shaped morphology with a post-synaptic density (PSD) at its tip. Modulation of the actin cytoskeleton drives these morphological changes as well as the spine dynamics that underlie learning and memory. Several PSD molecules respond to glutamate receptor activation and relay signals to the underlying actin cytoskeleton to regulate the structural changes in spine and PSD morphology. α-Actinin-2 is an actin filament cross-linker, which localizes to dendritic spines, enriched within the post-synaptic density, and implicated in actin organization. We show that loss of α-actinin-2 in rat hippocampal neurons creates an increased density of immature, filopodia-like protrusions that fail to mature into a mushroom-shaped spine during development. α-Actinin-2 knockdown also prevents the recruitment and stabilization of the PSD in the spine, resulting in failure of synapse formation, and an inability to structurally respond to chemical stimulation of the N-methyl-D-aspartate (NMDA)-type glutamate receptor. The Ca^2+^-insensitive EF-hand motif in α-actinin-2 is necessary for the molecule's function in regulating spine morphology and PSD assembly, since exchanging it for the similar but Ca^2+^-sensitive domain from α-actinin-4, another α-actinin isoform, inhibits its function. Furthermore, when the Ca^2+^-insensitive domain from α-actinin-2 is inserted into α-actinin-4 and expressed in neurons, it creates mature spines. These observations support a model whereby α-actinin-2, partially through its Ca^2+^-insensitive EF-hand motif, nucleates PSD formation via F-actin organization and modulates spine maturation to mediate synaptogenesis.

## Introduction

Actin filaments are the primary structural determinant of spines, and their remodeling in response to NMDA-receptor activation is critical for spine plasticity [1], [2]. Several genes encoding post-synaptic molecules that regulate the architecture of the actin cytoskeleton are mutated in neurodevelopmental disorders [3]–[6]. Thus, parsing the mechanisms that regulate actin filament organization in dendritic spines is crucial to understanding the cellular foundation of cognition.

Actin filament bundling by α-actinin is implicated in a variety of cellular structures such as stress fibers, adhesions, junctions, and dendritic spines [7], [8]. α-Actinin is an antiparallel homodimer with an actin-binding site on either end that mediates actin filament cross-linking [9]. The N-terminal actin binding domain is followed by four tandem spectrin repeats and a calmodulin-like domain, that determines each isoform's calcium sensitivity, at its C-terminus [10]–[12]. Although three of the four α-actinin isoforms, α-actinin-1, -2, and -4, have been identified in rat PSD fractions by mass spectrometry [13], [14] and RT-PCR of cultured hippocampal neurons [15], immunofluorescence and electron microscopy studies have shown specific enrichment of α-actinin-2 in the PSD of excitatory synapses in pyramidal neurons of the cortex and hippocampus [8], [16]–[19].

In addition to cross-linking actin filaments, α-actinin interacts with several membrane-associated proteins, including integrins, α-catenin, and the L-type Ca^2+^ channel Ca_v_1.2, and through these interactions α-actinin is thought to couple these molecules to actin filaments [7], [20]–[22]. *In vitro* binding assays suggest α-actinin-2 interacts directly with the NR1 and NR2B subunits of the NMDA receptor [16]. *In vitro* studies also suggest α-actinin-2 binds to densin-180 to form a ternary complex with CaMKIIα and NR2B [23]. These observations are supported by studies in HEK293 cells, in which α-actinin-2 targets CaMKIIα to F-actin and enhances the interaction between CaMKIIα and NR2B [24]. These putative interactions suggest α-actinin-2 could interpret signals and mediate interactions between PSD components and the actin cytoskeleton, and thus play a pivotal role in post-synaptic organization.

α-Actinin regulation and function in spines is poorly understood and relies largely on *in vitro* binding interactions or studies in non-neuronal cells. PtdIns(4,5)*P*_2_, PIP2, binds to the actin-binding domain of α-actinin-2 and tethers it to the plasma membrane, a function thought to maintain the open state of the NMDA receptor [25]. Neurons expressing an α-actinin-2 mutant unable to interact with PIP2 display significantly reduced peak and steady-state NMDA current compared to neurons expressing *wild-type* α-actinin-2 [25]. In one study, overexpression of α-actinin-2 increased the length and density of dendritic protrusions in cultured hippocampal neurons, suggesting a role in determining spine morphology [8].

To ascertain a biological function for α-actinin-2 in spines, we knocked down α-actinin-2 in hippocampal neurons via short interfering (si)RNA. We find that loss of α-actinin-2 increases spine density and the presence of filopodia-like spines that lack a PSD. These immature spines do not form synapses and therefore do not mature in response to chemical stimulation. We further show the Ca^2+^-insensitive EF-hand motif in α-actinin-2 is critical for its role in spine morphogenesis and PSD organization. Expression of either α-actinin-4 or a Ca^2+^-sensitive α-actinin-2 mutant does not rescue spine morphology and PSD assembly in neurons lacking endogenous α-actinin-2. However, expression of a Ca^2+^-insensitive α-actinin-4 mutant does rescue PSD organization. These studies suggest α-actinin-2 re-organizes the actin cytoskeleton in filopodia-like dendritic protrusions to promote assembly of the PSD and mediate its transition to a mature, mushroom-shaped morphology.

## Results

### α-Actinin-2 regulates spine morphology and density

**Figure 1:**
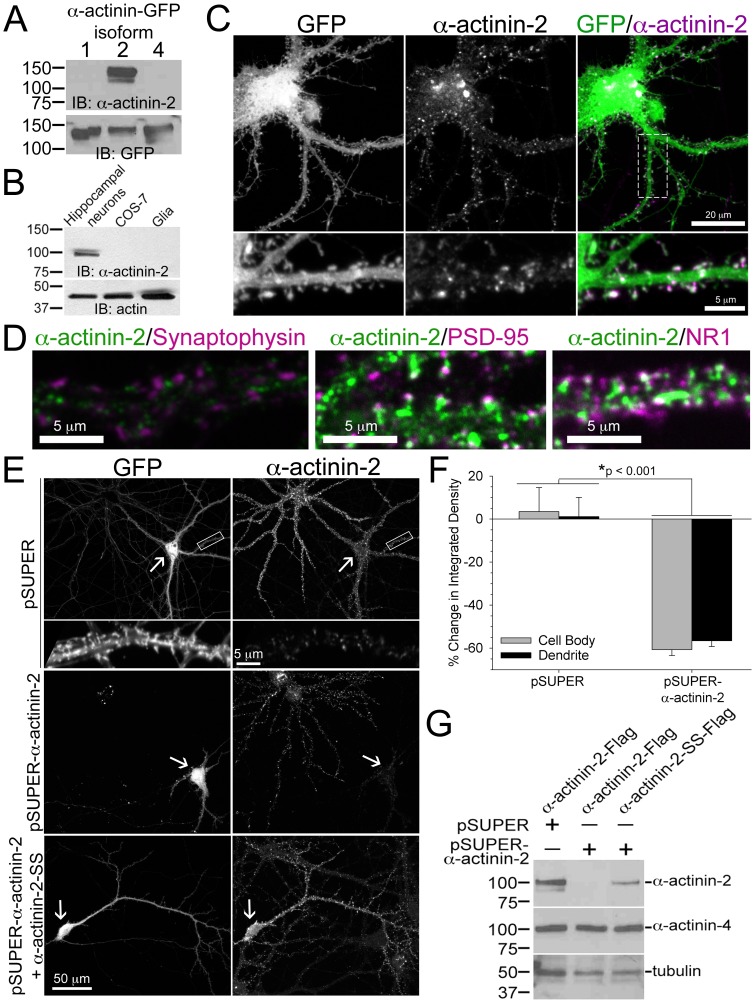
α-actinin-2 localizes to post-synaptic sites in dendritic spines on hippocampal neurons. **A)** An anti-α-actinin antibody (ab68167) recognizes α-actinin-2 and not α-actinin-1 or α-actinin-4. CHO-K1 cells were transfected with human α-actinin-1-GFP, α-actinin-2-GFP, or α-actinin-4-GFP. Cells were lysed and immunoblotted for α-actinin-2 and GFP. **B**) α-Actinin-2 is enriched in hippocampal neurons but not in glia cells or COS-7 cells, which lacks α-actinin-2. Cells were lysed and immunoblotted for α-actinin-2. Actin is the loading control. **C)** α-Actinin-2 localizes to dendritic spines. Hippocampal neurons were transfected at DIV 17 with GFP (green), and fixed, and immunostained for endogenous α-actinin-2 (magenta) at DIV 21. **D)** α-Actinin-2 co-localizes with post-synaptic markers, but not with a pre-synaptic marker. Hippocampal neurons were fixed at DIV 16 or 21 and immunostained for endogenous α-actinin-2 (green) and either endogenous synaptophysin, PSD-95, or the NR1 subunit of the NMDA receptor (magenta). **E**–**G)** The siRNA is specific for α-actinin-2. Hippocampal neurons were co-transfected at DIV 17 with GFP and either a control empty vector (pSUPER), or a vector containing siRNA against α-actinin-2 (pSUPER-α-actinin-2), or the α-actinin-2 siRNA-containing vector plus a α-actinin-2 vector conferring resistance to RNAi (pSUPER-α-actinin-2+ α-actinin-2-SS). The cells were fixed at DIV 21 and immunostained for endogenous α-actinin-2. Arrows point to the neurons co-expressing GFP and its immunostaining for α-actinin-2. For each condition (55 control cells and 46 α-actinin-2 knockdown cells), the integrated density of the cell body and dendrites were measured from the transfected neuron and adjacent untransfected neuron of the same image and the percent change was plotted, F. Error bars represent SEM. p-values were derived using the paired t-test. **G)** CHO-K1 cells were co-transfected with GFP, pSUPER or pSUPER-α-actinin-2, plus either α-actinin-2-Flag or α-actinin-2-SS-Flag. Transfection efficiency was close to 100% as >95% of the cells in each condition exhibited GFP fluorescence (data not shown). Cells were lysed 72 hours after transfection and immunoblotted for α-actinin-2 and α-actinin-4. Tubulin is the loading control.

Whereas previous studies show enrichment of α-actinin-2 in rat forebrain post-synaptic density fractions [13], [14]; many of the commercially available antibodies for α-actinin-2 cross-react with other highly homologous and equal-sized α-actinin isoforms. To clarify this issue, we used an anti-sarcomeric α-actinin antibody (ab68167) that is specific for α-actinin-2 and does not cross-react with α-actinin isoforms 1 and 4, which are present in CHO-K1 and COS-7 cells and rat forebrain PSD fractions [13], [14] (Figure 1A). Using this reagent, we find that α-actinin-2 is enriched in hippocampal neurons and is not present in the surrounding glia cells, which contain abundant levels of α-actinin-4 (Figures 1B and 7A). We also observed co-localization between α-actinin-2 and the post-synaptic protein, PSD-95, partial co-localization with the NR1 subunit of the NMDA receptor (Figure 1D), but no co-localization with the pre-synaptic molecule, synaptophysin, indicating α-actinin-2 is only enriched on the post-synaptic side of synapses (Figure 1D). These observations extend previous findings indicating that α-actinin-2 localizes to dendritic spines of hippocampal neurons [16], [17] (Figure 1C).

To determine the role of α-actinin-2 in dendritic spine morphogenesis, we knocked down endogenous expression of α-actinin-2 with siRNA expressed by the pSUPER vector, which drives constitutive expression of the siRNA in mammalian cells [26]. An ON-TARGETplus set of 4 siRNA sequences targeting rat α-actinin-2 were purchased from Dharmacon-Thermo Scientific and cloned into the pSUPER cassette (data not shown). Only one of these siRNA sequences knocked down endogenous α-actinin-2 protein levels without inducing off-target effects. This isoform-specific siRNA sequence targets α-actinin-2 mRNA, and 96 hrs after transfection endogenous α-actinin-2 immunofluorescence levels in the cell body and dendrites were decreased by an average of 60% in comparison to cells expressing the empty pSUPER vector (Figures 1E, F). Cells in the knockdown range of 40–90% were chosen for spine analysis. Co-expression of human α-actinin-2 with a silent mutation in a serine residue of the target sequence (α-actinin-2-SS), conferring resistance to RNA inhibition (RNAi), rescued expression (Figure 1E). Western blot of CHO-K1 cells expressing exogenous α-actinin-2 with the siRNA shows specific and near complete knockdown of α-actinin-2 without a decrease in α-actinin-4 expression levels (Figure 1G).

**Figure 2:**
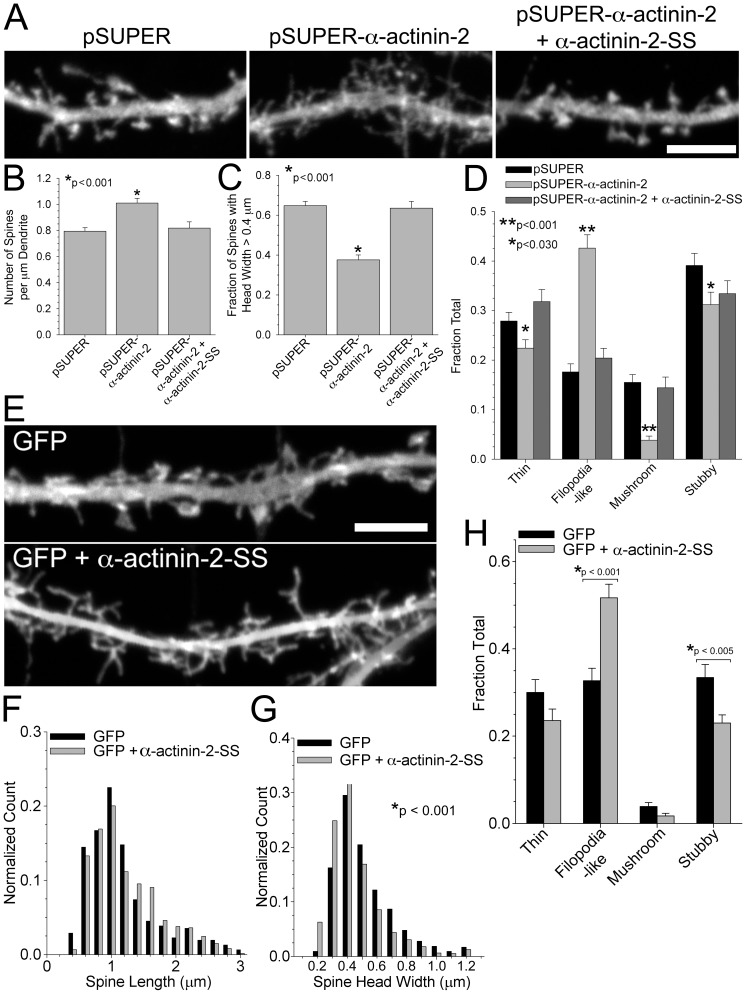
Knockdown of α-actinin-2 increases spine number and inhibits spine maturation. **A)** Hippocampal neurons were co-transfected at DIV 17 with GFP and either pSUPER, pSUPER-α-actinin-2, or pSUPER-α-actinin-2 plus α-actinin-2-SS. Neurons were fixed on DIV 21 and scored for **(B**–**D)** changes in spine density, head width, and morphology. Inhibition of α-actinin-2 at DIV 17 increases spine density, B. For each condition, spine density was analyzed on 35–42 dendrites from 3 separate cultures. The fraction of spine head widths >0.4 µm is significantly reduced in neurons with α-actinin-2 knocked down at DIV 17, C. α-Actinin-2 knockdown creates an increase in the fraction of filopodia-like spines and a decrease in the fraction of mushroom-shaped spines, thin spines (long protrusions with small head at tip), and stubby spines, D. For quantification of spine head width and morphology, 1444–2081 spines from 51 control neurons, 40 α-actinin-2 knockdown neurons, and 35 rescue neurons of 3 separate cultures were analyzed. **E)** Hippocampal neurons were transfected at DIV 6 with either GFP alone or GFP + α-actinin-2-SS. Neurons were fixed on DIV 22 and scored for **(F**–**H)** changes in spine length, head width, and morphology. Overexpression of α-actinin-2-SS increases spine length (F) and reduces spine head width (G). α-Actinin-2-SS overexpression creates an increase in the fraction of filopodia-like spines and a decrease in the fraction of stubby spines, H. For quantification of spine length, head width, and morphology, 311–610 spines from 15 control neurons and 21 α-actinin-2-SS overexpression neurons of 3 separate cultures were analyzed. Error bars represent SEM. p-values were derived using the paired t-test. Scale = 5 µm.

Knockdown of α-actinin-2 with siRNA at day *in vitro* (DIV) 17 inhibited spine maturation and increased the number of spines along the dendrites (Figures 2A, B). The spines on neurons with diminished α-actinin-2 expression were significantly thinner (Figures 2A, C). While control neurons exhibited many spines with a “mushroom” morphology, e.g. a large bulbous spine head on top of a short spine neck, neurons with α-actinin-2 knocked down displayed significantly fewer mushroom-shaped spines, and more headless, filopodia-like protrusions (Figures 2A, D). To show that this phenotype was specific for α-actinin-2, we co-transfected an RNAi-resistant α-actinin-2-SS with the siRNA plasmid and fixed the neurons 96 hours later. Spine density, spine head width, and the classic mushroom-shaped spine morphology, at later stages, e.g., DIV 21, were rescued by exogenous expression of α-actinin-2-SS (Figures 2A, B, C, and D).

Previous studies suggest that α-actinin-2 overexpression creates a spine morphology, e.g., increased density of longer and thinner spines [8], [27], that is similar to that in neurons lacking α-actinin-2. In agreement with these studies, we find that exogenous overexpression of α-actinin-2-SS in our hippocampal cultures increases spine lengths, reduces spine head widths, and creates an increased fraction of filopodia-like spine morphologies in comparison to control cells expressing only GFP (Figures 2E–H). Taken together, these findings suggest that an optimum amount of α-actinin-2 is required to maintain normal spine morphology.

**Figure 3:**
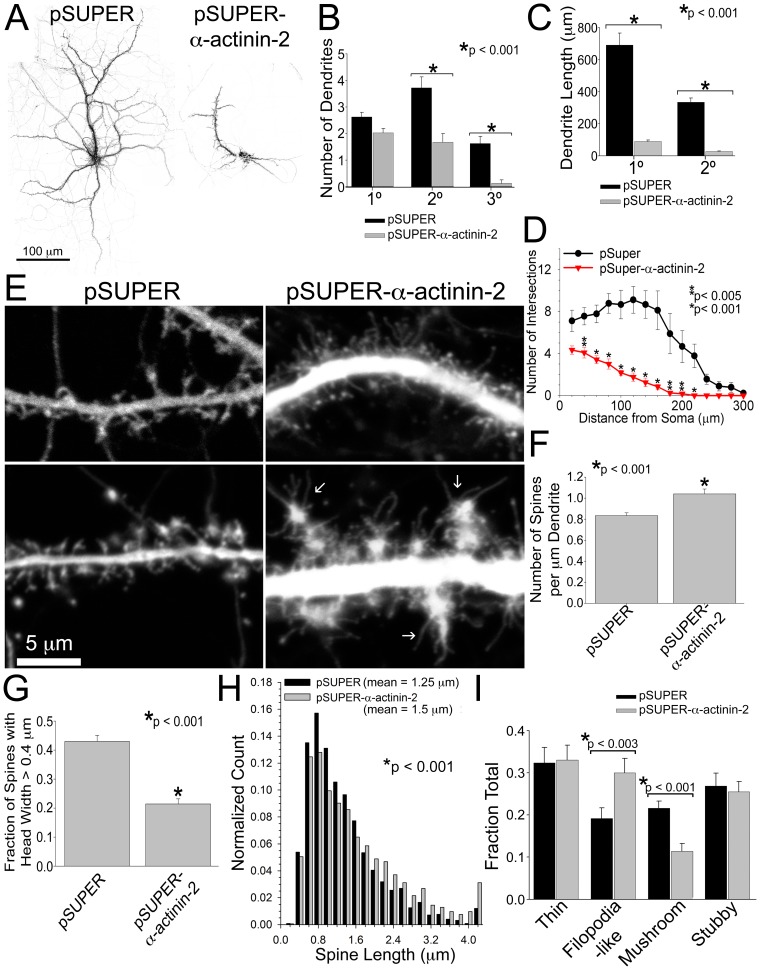
α-Actinin-2 contributes to development of dendritic arbors and spine morphology in early cultures. **A–C)** Hippocampal neurons were co-transfected at DIV 6 with GFP and either pSUPER or pSUPER-α-actinin-2 and fixed on DIV 22. Note the reduced size and number of dendrite arbors on neurons lacking α-actinin-2. While there is no difference in the number of primary dendrites, the number of secondary and tertiary dendrites on neurons lacking α-actinin-2 is reduced, B. The length of primary and secondary dendrites is smaller in neurons with α-actinin-2 knocked down, C. The branching complexity is reduced in neurons lacking α-actinin-2, D. Dendrites from 17 control neurons and 21 α-actinin-2 knockdown neurons from 2 different cultures were analyzed. **E)** Hippocampal neurons were co-transfected at DIV 6 with GFP and either pSUPER or pSUPER-α-actinin-2. Neurons were fixed on DIV 21 and scored for **(E**–**I)** changes in spine density, length, head width, and morphology. Two examples of control neurons and α-actinin-2 knockdown neurons are shown. Arrows point to irregularly shaped protrusions containing numerous filopodia, which is observed in several α-actinin-2 knockdowns. Inhibition of α-actinin-2 increases the number of spines per µm length of the dendrite (spine density), F. Spine density was quantified from 73 control neurons and 65 α-actinin-2 knockdown neurons from more than 3 cultures. The fraction of spine head widths >0.4 µm is significantly reduced in neurons with α-actinin-2 knocked down, G. Spine length is shifted to the right (longer) in neurons lacking α-actinin-2, H. α-Actinin-2 knockdown creates an increase in the fraction of filopodia-like spines (long protrusions without a spine head) and a concomitant decrease in the fraction of mushroom-shaped spines, I. For quantification of spine width, length, and morphology, 1875–2245 spines from 29 control neurons and 35 α-actinin-2 knockdown neurons from more than 3 cultures were analyzed. Error bars represent standard error of the mean (SEM). p-values were derived using the paired t-test (B, C, D, H, I) and Mann-Whitney test (F, G).

Inhibition of α-actinin-2 by siRNA knockdown at very early stages of neuron development, DIV 6, reduced the branching complexity and induced gross morphology changes in the dendrite arbors, including fewer secondary and tertiary dendrites (Figures 3A, B, D). The length of the primary and secondary dendrites was shorter in neurons lacking normal levels of α-actinin-2 (Figures 3A, C). This observation shows that α-actinin-2 is required for the proper growth of dendrites. Knockdown of α-actinin-2 at DIV 6 also increased the number of spines along dendrites and decreased spine head width and the fraction of mushroom-shaped spines, similar to the spine phenotype observed following knockdown of α-actinin-2 at DIV 17, when dendrite arbor growth is complete (Figures 3E–I). This shows that the immature spine phenotype produced by α-actinin-2 knockdown is not due to its effects on dendrite growth. Inhibiting α-actinin-2 at early stages also creates longer spines (Figures 3E, H). Although difficult to quantify, many of these filopodia-like protrusions on α-actinin-2 knockdown neurons appeared thinner and “hair-like” in contrast to the immature, filopodia-like spines on control neurons (Figure 3E). Additionally, irregularly shaped protrusions containing numerous filopodia (arrows) appeared on some dendrites of neurons lacking α-actinin-2 (Figure 3E). Using time-lapse confocal imaging, we found no difference in dynamics between aberrant protrusions on neurons lacking α-actinin-2 and the normal spines on control neurons, suggesting that α-actinin-2 does not regulate spine motility (data not shown). Taken together, these findings show that α-actinin-2 is necessary for the proper development of dendrites and spines.

### α-Actinin-2 is required for spine maturation in response to NMDA receptor stimulation

**Figure 4:**
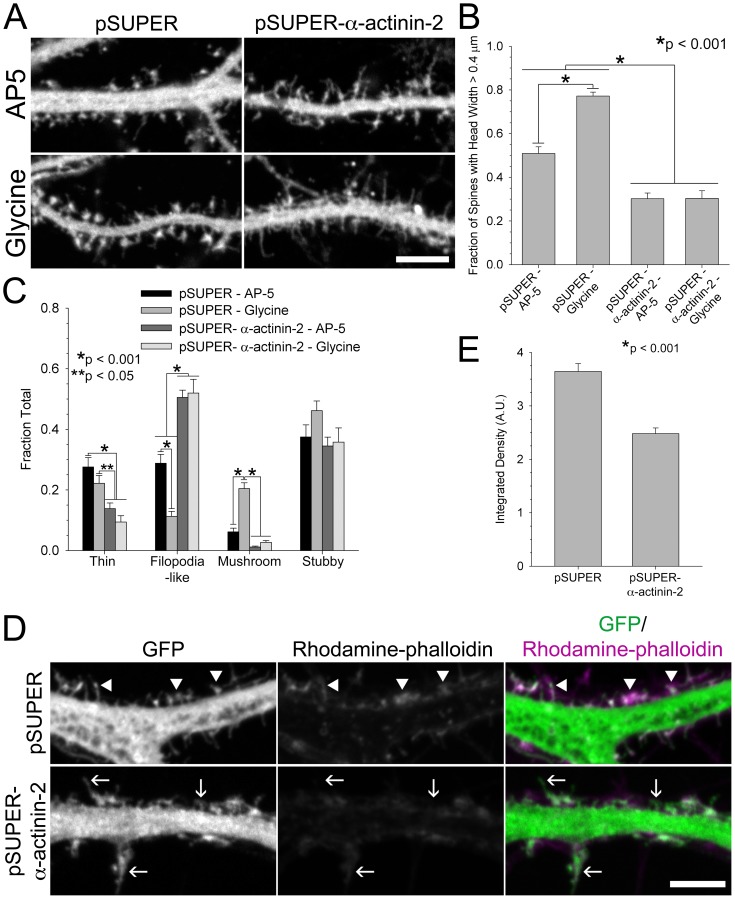
Knockdown of α-actinin-2 prevents spine maturation in response to NMDA receptor activation. **A)** When α-actinin-2 is knocked down, spines do not shorten or assume a “mushroom” morphology in response to glycine. Hippocampal neurons were co-transfected at DIV 6 with GFP and either pSUPER or pSUPER-α-actinin-2. Neurons were chronically treated with AP-5, an NMDA receptor antagonist, to inhibit spine maturation. At DIV 19–21, neurons were acutely stimulated by the addition of 200 µM glycine and AP-5 withdrawal, while control neurons were continuously treated with AP-5. **B**–**C)** Quantification of spine morphology in response to α-actinin-2 inhibition and glycine stimulation. Fraction of spines with a large head, spine tip width >0.4 µm, increases in response to glycine stimulation but is prevented by α-actinin-2 knockdown, B. In contrast to stimulated controls, inhibition of α-actinin-2 does not increase the fraction of mushroom-shaped spines and decrease filopodia-like spines, C. For each condition, 556–1721 spines of 16–24 neurons from 2 separate cultures were analyzed. **D**–**E)** α-Actinin-2 knockdown prevents enrichment of actin filaments in spines. Hippocampal neurons were co-transfected at DIV 6 with GFP and either pSUPER or pSUPER-α-actinin-2, fixed on DIV 21, and stained for rhodamine-phalloidin. Arrowheads mark actin enrichment in spines of control neurons and arrows point to the lack of actin in spines of neurons with α-actinin-2 knocked down, D. The fluorescent intensity of rhodamine-phalloidin is reduced in spines with α-actinin-2 knocked down, E. For each condition, 87–97 spines from 5 neurons were analyzed. Error bars represent SEM. p-values were derived using the paired t-test. Scale = 5 µm.

Since neurons lacking normal levels of α-actinin-2 showed an increased density of immature, filopodia-like protrusions that failed to develop into mushroom-shaped spines, we hypothesized that α-actinin-2 would be required for the acute, activity-induced spine morphology changes that occur in response to chemical stimulation [28], [29]. To test this, we selectively activated synaptic NMDA receptors with the co-agonist glycine [30], [31]. As expected, 20 min following brief treatment with glycine (200 µM for 3 min), control neurons displayed a significant increase in the fraction of spines with wider heads and mushroom-shaped spines when compared to unstimulated neurons maintained in bath solution containing the NMDA receptor antagonist, AP-5 (200 µM) (Figures 4A, B, C). In contrast, neurons with α-actinin-2 knocked down, under both conditions, continued to display the increased density of thinner, filopodia-like protrusions (Figures 4A, B, C). This demonstrates that α-actinin-2 is required for the transition to an enlarged, mushroom-shaped spine in response to NMDA receptor stimulation and corroborates our finding that α-actinin-2 is necessary for proper spine development.

NMDA receptor activation triggers post-synaptic signaling cascades that impact actin filament organization and spine maturation [1], [3], [32], [33]. The “hair-like” protrusions displayed on both stimulated and un-stimulated neurons lacking α-actinin-2, imply a misorganization of actin filaments in these immature spines. Using rhodamine-phalloidin, we visualized actin filaments in spines from control neurons and α-actinin-2 knock down neurons. Interestingly, we found that spines in neurons lacking α-actinin-2 were mostly devoid of detectable actin filament bundles, especially at the spine tip, in contrast to abundant phalloidin-bound actin filaments visible in spines of control neurons (Figures 4D, E). This finding suggests that α-actinin-2, likely through its actin cross-linking activity is needed to produce detectable actin filament bundles in the spine, which in turn drives structural changes underlying spine maturation.

### α-Actinin-2 is required for synapse formation

**Figure 5:**
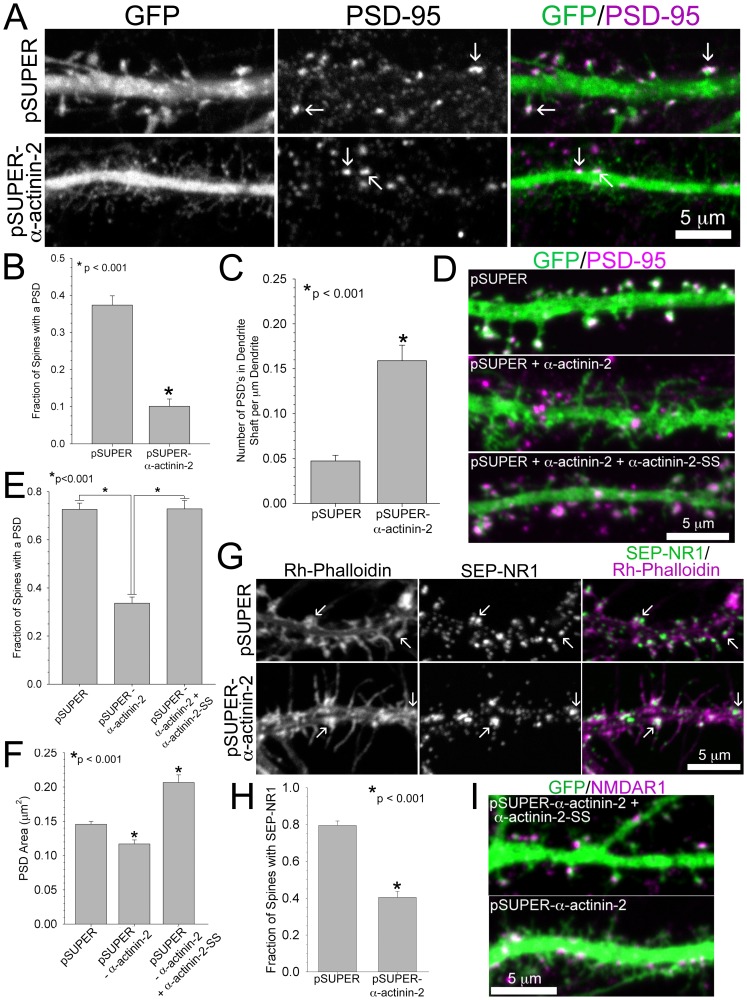
Loss of α-actinin-2 prevents assembly of the post-synaptic density. **A–C)** α-Actinin-2 knockdown inhibits PSD assembly in spines. Hippocampal neurons were co-transfected at DIV 6 with GFP and either pSUPER or pSUPER-α-actinin-2, fixed on DIV 21, and immunostained for PSD-95. Arrows mark localization of PSD-95 in spines or in the dendrite shaft, A. The fraction of spines with localized PSD-95 is reduced in neurons with α-actinin-2 knocked down, B. PSD-95 localizes to the dendrite shaft with increased frequency in neurons lacking α-actinin-2, C. For each condition, 37-45 neurons from 5 separate cultures were analyzed. **D**–**F)** Hippocampal neurons were co-transfected at DIV 17 with GFP and either pSUPER, pSUPER-α-actinin-2, or pSUPER-α-actinin-2 plus α-actinin-2-SS, D. The fraction of spines with PSD-95 is rescued in neurons expressing exogenous α-actinin-2-SS, E. The area of PSD-95 is significantly reduced in spines lacking α-actinin-2 and rescued in neurons expressing exogenous α-actinin-2-SS, F. For each condition, PSD-95 area was measured from 543–991 spines of 23–27 neurons from 3 separate cultures. **G**–**I)** α-Actinin-2 knockdown prevents the recruitment of the NMDA receptor to the spine. Hippocampal neurons were co-transfected at DIV 6 with SEP-NR1 and either pSUPER or pSUPER-α-actinin-2, fixed on DIV 22, and immunostained for GFP and rhodamine-phalloidin. Arrows mark SEP-NR1 localization in either spines or in the dendrite, G. The fraction of spines co-localized with SEP-NR1 is reduced in neurons with α-actinin-2 knocked down, H. For each condition, 12–19 neurons from 3 separate cultures were analyzed. **I)** Hippocampal neurons were co-transfected at DIV 17 with GFP and either pSUPER-α-actinin-2 or pSUPER-α-actinin-2 plus α-actinin-2-SS, fixed on DIV 21, and immunostained for NMDAR1. Error bars represent SEM. p-values were derived using the paired t-test.

The morphological changes associated with spine maturation and proper arrangement of post-synaptic molecules, which nucleate a signaling platform to orchestrate the structural response to NMDA receptor activation, require the spine to be in stable contact with a potentiated pre-synaptic bouton [34]–[36]. PSD-95 is a key molecule in PSD organization and synaptic plasticity; it is also a good marker for PSD location and organization, since it is found in synapses at early stages [37]. To address whether α-actinin-2 contributes to PSD organization, we immunostained for PSD-95 in DIV 21 control cells and age-matched neurons with α-actinin-2 knocked down at DIV 6–9. In contrast to control neurons, in which PSD-95 was observed in most spines, the spines of neurons with diminished levels of α-actinin-2 lacked detectable, organized PSD-95 (Figures 5A, B). In these neurons, PSD-95 only localized to the dendrite shaft (Figures 5A, C). Loss of α-actinin-2 during mid-development, DIV16-19, when many spines have established connections with a pre-synaptic bouton, induced an increased density of immature spines that also lack PSD-95 and reduced the overall size of any pre-existing PSD in spines (Figures 5D, E, F). This suggests that α-actinin-2 is not only required for the recruitment of post-synaptic molecules, but it is also required for the maintenance of the PSD. Importantly, co-expression of α-actinin-2-SS rescues PSD-95 localization and size in dendritic spines (Figures 5D, E, F); corroborating our finding that α-actinin-2 is required for PSD assembly in the spine. In agreement with previous studies [8], overexpression of α-actinin-2-SS also increases the density of immature spines that lack PSD-95 (data not shown), indicating a requirement for normal synaptic amounts of α-actinin-2 to mediate PSD assembly in the spine.

Since PSD-95 interacts with the NR1 subunit of NMDA receptors [38] and α-actinin-2 directly binds to NR1 *in vitro* [16], [39], we asked whether the NMDA receptor formed discernable structures at the tips of spines of neurons lacking α-actinin-2. We co-expressed the ubiquitous NR1 subunit of the NMDA receptor fused to a super-ecliptic pHluorin (SEP-NR1), which displays GFP fluorescence at the membrane surface when SEP is exposed to a neutral environment [29]. Rhodamine-phalloidin was used to visualize actin-rich spines. While discrete clusters of SEP-NR1 were seen in the spines of control neurons, they did not localize to detectable phalloidin-bound protrusions in neurons with α-actinin-2 knocked down (Figures 5G, H). Instead, SEP-NR1 clustered within the dendrite shaft at the base of some filopodia-like protrusions (Figures 5G). Notably, clusters of actin filaments co-localized with SEP-NR1 (Figure 5G), suggesting that NR1 localizes to actin-rich sites and diminished actin filament bundles in spines of neurons lacking α-actinin-2 prevent its recruitment to spine tips. Importantly, endogenous NR1 subunit of NMDA receptor (NMDAR1) also mis-localized to the dendrite shaft in neurons with α-actinin-2 knocked down during mid-development (DIV 17), and co-expression of α-actinin-2-SS rescues NMDAR1 localization in spines (Figure 5I). These results indicate α-actinin-2 organizes the actin filaments in the spine to recruit and assemble key components of the PSD, including PSD-95 and NMDA receptors.

**Figure 6:**
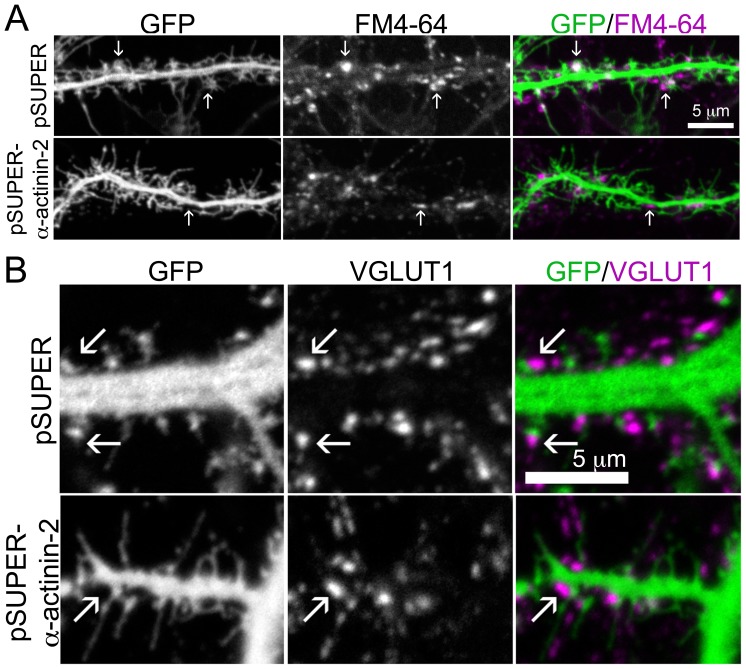
Loss of α-actinin-2 prevents synapse formation. **A)** Actively firing pre-synaptic boutons do not synapse with spines on neurons lacking α-actinin-2. Hippocampal neurons were co-transfected at DIV 6 with GFP and either pSUPER or pSUPER-α-actinin-2, treated with FM4-64 for 5 min on DIV 19 and observed live. Arrows mark FM4-64 juxtaposition to spines or the dendrite. The fraction of spines juxtaposed to FM4-64 is reduced in neurons lacking α-actinin-2 (see results). For each condition, 14 neurons from 2 separate cultures were analyzed. **B)** Loss of α-actinin-2 prevents synapse formation with excitatory axon boutons. Hippocampal neurons were co-transfected at DIV 6 with GFP and either pSUPER or pSUPER-α-actinin-2, fixed on DIV 21, and immunostained for VGLUT1. Arrows mark VGLUT1 juxtaposition to spines or the dendrite. The fraction of spines juxtaposed to VGLUT1 is reduced in neurons lacking α-actinin-2 (see results). For each condition 23–26 neurons from 2 separate cultures were analyzed.

To determine whether excitatory, pre-synaptic boutons could synapse with spines of neurons lacking α-actinin-2 and an organized PSD, we briefly exposed the neurons to the lipophilic styryl dye, FM4-64, which is endocytosed within recycled synaptic vesicles, thereby marking synapses actively releasing neurotransmitters [40]. We observed significantly fewer spines co-localized with FM4-64 puncta in neurons lacking α-actinin-2 than in control neurons (9% of spines juxtaposed to FM4-64 in control neurons versus 0.2% of spines in neurons lacking α-actinin-2, p<0.001, paired t-test) (Figures 6A). Despite the significant difference in FM4-64 labeling between control neurons and neurons with α-actinin-2 knocked down, we were surprised by the small fraction of FM4-64 puncta detected in control neurons using our culture system. Therefore, we immunostained the neurons for VGLUT1, an excitatory pre-synaptic marker, and found that VGLUT1 was also not apposed to many spines in neurons with α-actinin-2 knocked down (Figures 6B). In contrast, VGLUT1 apposed most spines of control neurons (66.1% of spines juxtaposed to VGLUT1 in control neurons versus 43.5% of spines in α-actinin-2-deficient neurons, p<0.002, paired t-test) (Figures 6B). These findings show that immature dendritic protrusions of neurons deficient in α-actinin-2 do not form organized synapses with potentiated axons and lack functional components of the PSD. The absence of a functional synapse suggests a likely explanation for why spines lacking α-actinin-2 do not mature morphologically in response to chemical stimulation.

**Figure 7:**
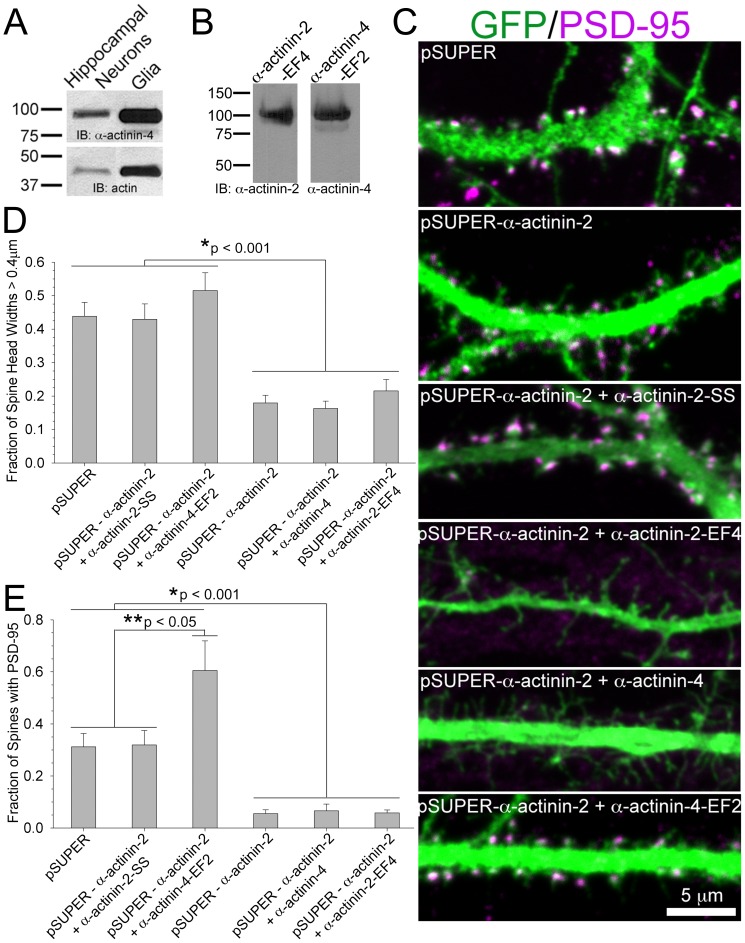
The isoform-specific α-actinin-2 EF hand domain dictates normal spine morphology and PSD assembly. **A)** α-Actinin-4 is ubiquitously enriched within hippocampal neurons and glia cells. Cells were lysed and immunoblotted for α-actinin-4. Actin is the loading control. **B)** α-Actinin-2-EF4 and α-actinin-4-EF2 run at 100KDa, equivalent to endogenous *wild type* α-actinin-2 and α-actinin-4, respectively. **C)** The calcium-insensitive EF hand domain in α-actinin-2 dictates normal spine morphology and PSD organization. Hippocampal neurons were co-transfected at DIV 17 with GFP and either pSUPER, pSUPER-α-actinin-2, pSUPER-α-actinin-2 plus α-actinin-2-SS, pSUPER-α-actinin-2 plus α-actinin-2-EF4, pSUPER-α-actinin-2 plus α-actinin-4, or pSUPER-α-actinin-2 plus α-actinin-4-EF2. Neurons were fixed on DIV 21 and scored for changes in head width **(D)** and fraction of spines with PSD-95 (**E**). For each condition, 305-1725 spines from 22-33 neurons of 3 separate cultures were analyzed. Error bars represent SEM. p-values were derived using the paired t-test.

### An isoform-specific domain dictates the function of α-actinin-2 in spines

α-Actinin-2 differs from α-actinin-4, which is also detected in hippocampal neuronal lysates (Figure 7A) and present in PSD fractions [13], [14]; it's EF-hand domain has several missing amino acids, rendering α-actinin-2 insensitive to calcium. To determine whether this Ca^2+^-insensitive domain is critical for the function of α-actinin-2, we swapped the EF-hand of α-actinin-2 with the Ca^2+^-sensitive EF-hand of α-actinin-4, and vice-versa. Western blot confirmed that the Ca^2+^-sensitive α-actinin-2 mutant (α-actinin-2-EF4) and the Ca^2+^-insensitive α-actinin-4 mutant (α-actinin-4-EF2) ran at the same size as *wild type* α-actinin-2 and α-actinin-4, respectively (Figure 7B). In contrast to rescue by *wild type* α-actinin-2, co-expression of α-actinin-2-EF4 with the α-actinin-2 siRNA did not rescue spine morphology and assembly of the PSD in spines, indicating a requirement for the Ca^2+^-insensitive EF hand domain (Figure 7C–E). Exogenous human α-actinin-4 was also unable to rescue spine morphology and PSD assembly when co-expressed with the α-actinin-2 siRNA, displaying a spine morphology phenotype similar to that of α-actinin-2-EF4 (Figure 7C–E). Interestingly, expression of the Ca^2+^-insensitive α-actinin-4-EF2 mutant rescued PSD organization and spine morphology, suggesting that the Ca^2+^-insensitive EF hand domain from α-actinin-2 dictates PSD assembly and spine morphology (Figures 7C–E). These findings show that the Ca^2+^-insensitive EF hand domain in α-actinin-2 regulates its function in dendritic spines, and that α-actinin-2 has an isoform-specific, non-redundant role in dictating spine morphology and assembly of post-synaptic proteins.

## Discussion

The actin cross-linking protein, α-actinin, plays a central role in organizing actin filaments in various cellular locations, including stress fibers [41], the lamellipodia of migrating cells [42], cell-matrix adhesions [43], cadherin-based cell-cell junctions [20], glomerular podoctyes [44], and neuronal synapses [7], [17]. In addition to cross-linking actin, α-actinin interacts with various transmembrane proteins, linking them to actin filaments, and with signaling complexes in actin-rich regions like adhesions [7]. Within excitatory hippocampal neurons, it is now clear from our work that α-actinin-2 serves to assemble key components of the PSD in dendritic spines, regulate synaptogenesis, and promote spine maturation.

Dendritic spines develop from exploratory, filopodial-like processes that protrude from the dendrite. In response to adhesive contact with and synaptic input from the pre-synaptic axon, these immature protrusions morph into a mature, mushroom-shaped structure [45]. Our results show that α-actinin-2 mediates spine maturation, as inhibition of α-actinin-2 causes a greater density of immature, filopodia-like protrusions that fail to mature into mushroom-shaped spines. Interestingly, while α-actinin-2 is dispensable for the growth of filopodia-like spines emanating from the dendrite shaft, α-actinin-2 is necessary for the growth of dendrites, as early knockdown of α-actinin-2 stunts the length and number of dendrites. Therefore, in addition to regulating spine maturation, α-actinin-2 also plays a significant role in supporting the growth of dendrite arbors.

The number of PSD-95 molecules determines the size and strength of the synapse and is required for stabilization of the synapse following synaptic activity [37], [46], [47]. Our results demonstrate that α-actinin-2 is required for PSD assembly and maintenance. We found that inhibiting α-actinin-2 during early spine development prevents PSD formation, whereas inhibiting during mid-development causes loss of the PSD within the spine. Presumably, an increased interaction between α-actinin-2 and actin filament bundles recruits additional actin bundles in the spine. Increased actin cross-linking could also serve to cluster the myriad of PDZ- and LIM-containing proteins in the PSD, recruit other actin-binding proteins to the PSD and thereby promote its enlargement [48]. An additional mechanism for recruitment of PSD molecules to the spine via α-actinin-2 could occur through its putative binding interactions with components of the PSD, including densin-180, CaMKIIα, and the NR1 and NR2B subunits of the NMDA-type glutamate receptor [16], [23]. Therefore, α-actinin-2 may nucleate assembly and growth of the PSD through direct recruitment of PSD molecules, and connect these proteins to actin filaments.

It is possible that increased stability of the PSD, which reinforces trans-synaptic connections, is required for spine maturation [34], [37], [49], [50]. Some observations support this hypothesis. Spines lacking α-actinin-2 do not appose excitatory, pre-synaptic boutons, as shown by the lack of VGLUT1 and FM4-64 juxtaposed to these immature spines. The absence of a functional synapse illustrates why glycine stimulation is insufficient in driving maturation of spines deficient in α-actinin-2.

Both knockdown and overexpression of α-actinin-2 induce similar phenotypes, consisting of an immature spine morphology lacking an organized PSD [8], [27]. Neurons deficient in α-actinin-2 have diminished actin filament bundles in their spines, whereas overexpression of α-actinin-2 in neurons likely creates spines with overly cross-linked actin filaments. Others have reported analogous observations. Knockout of the gene encoding the actin crosslinker protein spinophilin/neurabin II increased spine density *in vivo* and the number of filopodia-like protrusions in cultured neurons [53]. Furthermore, overexpression of other actin crosslinkers, including drebrin and a non-contractile myosin IIB mutant (MIIB R709C), increased spine length and the number of immature dendritic protrusions [51], [52]. These findings suggest that a fine balance of actin filament bundling in the spine is necessary to drive proper synapse maturation and spine morphology.

It is particularly interesting that α-actinin-2, a Ca^2+^-insensitive isoform well known for it role in striated muscle, is also enriched within dendritic spines. The significance of this is illustrated by our finding that a Ca^2+^-sensitive α-actinin-2 mutant cannot rescue spine morphology or PSD assembly in neurons lacking endogenous α-actinin-2, whereas a Ca^2+^-insensitive α-actinin-4 mutant can. Calcium binding to non-muscle α-actinin isoforms (1 and 4) reduces their binding affinity for actin, a major mechanism in regulating the biological activity of these isoforms in migrating cells [54]. Thus, α-actinin-2 may serve a unique function in dendritic spines to organize actin filaments and scaffold PSD molecules to the actin cytoskeleton despite high concentrations of calcium. In support of this view, calcium influx is thought to be mediated by the NMDA receptor, which α-actinin-2 couples to the actin cytoskeleton and Ca^2+^-sensitive α-actinin isoforms cannot maintain the NMDA receptor in an active, open state *in vitro* [39]. Therefore α-actinin-2 can stabilize components of the PSD to the actin cytoskeleton in Ca^2+^-rich compartments, such as dendritic spines.

We found that spines in neurons lacking α-actinin-2 are largely devoid of detectable actin filament bundles, suggesting that α-actinin-2 helps to form a template for the hierarchical addition of PSD molecules within the spine. This deduction is analogous to the role of α-actinin-1 in organizing actin filaments for the maturation of matrix adhesions and α-actinin-4-mediated assembly of E-cadherin junctions [43], [55].

In summary, the data presented here provide new insights into an actin-associated mechanism for PSD assembly in dendritic spines. We show that α-actinin-2 is a critical component of the actin machinery that regulates synaptogenesis and spine maturation, and the Ca^2+^-insensitive EF domain determines its molecular function in neurons. The loss of function data for α-actinin-2, provided here, serves as a framework for which future studies can build upon to elucidate the regulatory mechanisms by which α-actinin-2 determines spine morphology and nucleates PSD assembly, which is critical to synaptic plasticity.

## Materials and Methods

### Antibodies and reagents

α-Actinin-2 polyclonal antibody was obtained from Epitomics (Abcam68167) and used at a ratio of 1∶100. Postsynaptic density-95 (PSD-95) monoclonal antibody, used at a ratio of 1∶100 for immunostaining, and synaptophysin monoclonal antibody, used at a ratio of 1∶1000 for immunostaining, were purchased from Santa Cruz Biotechnology (Santa Cruz, CA). A GFP polyclonal antibody was obtained from Invitrogen (Carlsbad, CA) and used at a ratio of 1∶250. VGLUT1 monoclonal antibody was purchased from Synaptic Systems (Goettingen, Germany) and used at a ratio of 1∶2000. NMDAR1 monoclonal antibody, used at a ratio of 1∶100 for immunostaining, was purchased from Millipore (Billerica, MA). α-Actinin-4 polyclonal antibody was obtained from Proteintech (Chicago, IL) and used at a ratio of 1∶1000 for immunoblotting. Tubulin monoclonal antibody and actin monoclonal antibody, both used at a ratio of 1∶1000 for immunoblotting, were purchased from Sigma (St. Louis, MO) and Santa Cruz Biotechnology, respectively. Secondary anti-mouse, anti-rabbit, and anti-guinea pig antibodies conjugated to Alexa488, 568 and 647 were from Invitrogen. Tetrodotoxin and strychnine were purchased from Sigma and reconstituted in dH_2_O. AP-5 was purchased from Tocris Bioscience (Ellisville, MO) and reconstituted in dH_2_O. Rhodamine phalloidin was purchased from Cytoskeleton (Denver, CO) and used at a ratio of 1∶100. FM4-64FX was purchased from Life Technologies (Carlsbad, CA).

*Plasmids*. Human α-actinin-2-GFP was obtained from Origene (Rockville, MD) and cloned into a GFP-N1 vector via EcoRI and BsrG1, which cuts out the GFP sequence. α-actinin-1-GFP was described previously [56]. Human α-actinin-4-GFP was a gift from Martin Pollak (Beth Israel Deaconess Medical Center and Harvard Medical School, Boston, MA) and cloned into a GFP-N1 vector via XhoI and EcoRI to create α-actinin-4-GFP-N1 and also cloned into GFP-N1 via EcoRI and BsrG1. α-actinin-2-EF4 and α-actinin-4-EF2 were created by cloning three PCR fragments: α-actinin-2_nt1-2268_-EcoRI-α-actinin-4_nt2307-2514_-Kpn1-α-actinin-2_nt2466-2682_ and α-actinin-4_aa1-2304_-EcoRI-α-actinin-2_aa2271-2463_-Kpn1-α-actinin-4_aa2517-2733_. An ON-TARGETplus set of 4 siRNA sequences targeting rat α-actinin-2 were purchased from Dharmacon-Thermo Scientific and cloned into the pSUPER cassette according to the vector manufacturer's instructions (Oligoengine). The oligonucleotide ATGAGAGGCTAGCGAGTGA, corresponding to nucleotides 938 – 956 of rat α-actinin-2 knocked down endogenous rat α-actinin-2 and exogenous human α-actinin-2. siRNA-insensitive α-actinin-2 was generated by site-directed mutagenesis (Quickchange kit, Stratagene) introducing one silent mutation (AGT to TCC: Ser to Ser) in the RNAi target region of human α-actinin2, which shares 100% homology with rat. pC1-SEP-NR1 was a gift from Robert Malinow, Addgene plasmid 23999 [29].

### Neuronal culture and transfection

Low-density hippocampal cultures were prepared from E19 rat embryos as described previously [57]. All experiments were carried out in compliance with the Guide for the Care and Use of Laboratory Animals of the National Institutes of Health and approved by the University of Virginia Animal Care and Use Committee (Protocol Number: 2884). Neurons were plated on glass coverslips coated with 1 mg/ml poly-L-lysine at an approximate density of 70 cells/mm^2^ and were transfected using either a modified calcium phosphate precipitation method as described previously [57] or lipofection with lipofectamine 2000 (Life Technologies) used at a ratio of 2 µl lipofectamine 2000 per 1 µg DNA. pSUPER-α-actinin-2 was used in 3∶1 excess to GFP and the rescue construct to ensure knockdown in fluorescence-positive cells. Coverslips were flipped in their original dish with neurons facing up and the lipofectamine/DNA complexes were pipetted directly onto the neurons. The coverslips were moved 24 hours after transfection to a new glia-feeder layer cultured for an equal amount of time as the original glia-feeder layer. For neurons that were transfected via lipofection, 100 µM AP-5 was added at DIV 6. For the chemical stimulation experiments (Figure 4), neurons were chronically treated with 100 µM of the NMDA receptor antagonist, AP-5, from DIV 6-21 to inhibit NMDA receptor activation and attenuate spine maturation. Neurons were removed from the glia-feeder layer and placed in 1× Mg^2+^-free extracellular solution containing 15 mM NaCl, 0.5 mM KCl, 0.2 mM CaCl_2_, 3 mM glucose, 1 mM Hepes, 0.5 µM tetrodotoxin, and 1 µM strychnine, pH7.4 [30]. Neurons were then stimulated by AP-5 withdrawal and the addition of 200 µM glycine, incubated at 35°C, 5% CO_2_ for 3 min, while control neurons continued in the presence of AP-5 (200 µM). The solution was then removed and replaced with 1× Mg^2+^-free extracellular solution with tetrodotoxin and strychnine and incubated at 35°C, 5% CO_2_ for 20 minutes before fixation, as described by others [58], [59].

### CHO-K1 cell culture and transfection

CHO-K1 cells were cultured in standard conditions and transfected using lipofectamine (Life Technologies) at a ratio of 5 µl lipofectamine per 1 µg DNA. pSUPER-α-actinin-2 was used in 5∶1 excess to the Flag-tagged α-actinin-2 construct to ensure knockdown (Figure 1G). For western blot analysis, cells were lysed in RIPA buffer + protease inhibitor (Sigma).

### Immunocytochemistry

Neurons were fixed in PBS containing 4% formaldehyde, methanol-free, ultra-pure EM grade (Polysciences, Inc., Warrington, PA) with 4% sucrose for 20 min at room temperature and permeabilized with 0.2% Triton X-100 for 10 min. Alternatively, for PSD-95 and α-actinin-2 immunostaining, neurons were simultaneously fixed and permeabilized in 2% formaldehyde with 4% sucrose for 10 min at room temperature followed by cold methanol for 8 min at -20°C. After blocking with 20% goat serum/PBS for one hour at room temperature, the neurons were incubated with the appropriate antibodies in 5% goat serum/PBS for one hour at 37°C. Alternatively, for surface immunostaining of SEP-NR1, neurons were permeabilized after incubation with the primary and secondary antibodies, just prior to incubation with rhodamine-phalloidin. Coverslips were mounted with Vectashield mounting media (Vector Laboratories, Burlingame, CA). For FM4-64 labeling, neurons were removed from the glia-feeder layer and placed in high K^+^ HBS supplemented with 10 µM FM4-64, 1 mM tetrodotoxin, and 2.5 nM AP-5 for 90 seconds, as described by others [60]. Cells were then washed twice for 4 min in HBS and imaged live.

### Imaging and analysis

Confocal images were collected on an Olympus Fluoview 1000 microscope (IX81 base) equipped with a 60X/1.35 NA (oil) UPLSAPO 60X objective (Olympus). Green probes (GFP and Alexa488) were excited using the 488 nm laser line of a multi-Ar laser; red probes (Rhodamine and Alexa568) were excited with the 543 nm laser line of a He-Ne laser; the far-red probe Alexa647 was excited with the 635 nm line of an LD laser. Fluorescence emission was collected using the following dichroic mirror/filter combinations: SDM560/BA505-525 (GFP), SDM640/BA560-620 (Alexa568 and RhodamineX) and BA655-755 (Alexa647). Two-color fluorescence images of Alexa488 (GFP)/Alexa568 (RhodamineX) were collected in a Z-stack and in sequential mode. Images were acquired using Fluoview software (Olympus). For experiments involving integrated density analysis, figures 1E–F and 4D–E, imaging parameters were held constant for all images acquired. Spine number, length, width, integrated density, and PSD-95 area were quantified using Image J software. Any neurons exhibiting features of cell death, including blebbing of the dendrites, were excluded from analysis; among the population analyzed, the phenotypes persisted beyond the day chosen for analysis. Statistical analysis was performed using Sigma Plot 11. A non-parametric Mann-Whitney test was used to confirm all conclusions. Spine morphologies were defined as either filopodia-like, thin, mushroom, or stubby [61]: Filopodia-like spines are long and thin without a spine head, whereas thin spines contain a small head at the spine tip. Mushroom-shaped spines are shorter with a large spine head atop a neck. Stubby spines are short protrusions, either thin or wide, with no discernable neck.
